# Melanoregulin, Product of the *dsu* Locus, Links the BLOC-Pathway and Oa1 in Organelle Biogenesis

**DOI:** 10.1371/journal.pone.0042446

**Published:** 2012-09-11

**Authors:** Rivka A. Rachel, Kunio Nagashima, T. Norene O'Sullivan, Laura S. Frost, Frank P. Stefano, Valeria Marigo, Kathleen Boesze-Battaglia

**Affiliations:** 1 Neurobiology, Neurodegeneration & Repair Laboratory, National Eye Institute, Bethesda, Maryland, United States of America; 2 Frederick National Laboratory for Cancer Research, SAIC-Frederick, Frederick, Maryland, United States of America; 3 National Cancer Institute-Frederick, Frederick, Maryland, United States of America; 4 Department of Biochemistry, University of Pennsylvania, Philadelphia, Pennsylvania, United States of America; 5 Live Cell Imaging Core, School of Dental Medicine University of Pennsylvania, Philadelphia, Pennsylvania, United States of America; 6 Department of Biomedical Sciences, University of Modena and Reggio Emilia, Modena, Italy; Radboud University Nijmegen Medical Centre, The Netherlands

## Abstract

Humans with Hermansky-Pudlak Syndrome (HPS) or ocular albinism (OA1) display abnormal aspects of organelle biogenesis. The multigenic disorder HPS displays broad defects in biogenesis of lysosome-related organelles including melanosomes, platelet dense granules, and lysosomes. A phenotype of ocular pigmentation in OA1 is a smaller number of macromelanosomes, in contrast to HPS, where in many cases the melanosomes are smaller than normal. In these studies we define the role of the *Mreg^dsu^* gene, which suppresses the coat color dilution of Myo5a, melanophilin, and Rab27a mutant mice in maintaining melanosome size and distribution. We show that the product of the *Mreg^dsu^* locus, melanoregulin (MREG), interacts both with members of the HPS BLOC-2 complex and with Oa1 in regulating melanosome size. Loss of *MREG* function facilitates increase in the size of micromelanosomes in the choroid of the HPS BLOC-2 mutants *ruby*, *ruby2*, and *cocoa*, while a transgenic mouse overexpressing melanoregulin corrects the size of retinal pigment epithelium (RPE) macromelanosomes in *Oa1^ko/ko^* mice. Collectively, these results suggest that MREG levels regulate pigment incorporation into melanosomes. Immunohistochemical analysis localizes melanoregulin not to melanosomes, but to small vesicles in the cytoplasm of the RPE, consistent with a role for this protein in regulating membrane interactions during melanosome biogenesis. These results provide the first link between the BLOC pathway and Oa1 in melanosome biogenesis, thus supporting the hypothesis that intracellular G-protein coupled receptors may be involved in the biogenesis of other organelles. Furthermore these studies provide the foundation for therapeutic approaches to correct the pigment defects in the RPE of HPS and OA1.

## Introduction

These studies define the role of the *Mreg^dsu^* gene [1], which suppresses the coat color dilution of Myo5a, melanophilin [2], and Rab27a [3] mutant mice in maintaining melanosome size and distribution. Analysis of cellular abnormalities in patients and mice with HPS reveals that these genes function in biogenesis of lysosome-related organelle complexes (BLOCS) to mediate vesicle budding and transport from the trans-Golgi network to nascent organelles [4], [5]. In OA1, defects are limited to melanosomes, and pathological features are primarily restricted to the eye [6]. Findings suggest that OA1 may function as an intracellular G-protein coupled receptor regulating both melanosome size and maturation [7]. Indeed, Gαi3 has recently been identified as the first downstream component in Oa1 signaling in RPE melanosomes [8]. Individuals with either OA1 or HPS display visual system defects common to albinism, including reduced visual acuity and loss of stereoscopic vision from misrouting of retinal axons at the optic chiasm [7].

Genetic observations of natural mutants, including coat color in mouse, continue to yield a wealth of information about genes involved in many processes, including vesicle transport and organelle formation. Many of these mouse coat color mutants also display abnormalities of other systems, such as the platelet dense granule defects in HPS. Melanoregulin loss of function, by contrast, presents with no observable coat color phenotype, and the *dilute suppressor* (*dsu*) mutation was originally identified as an unlinked suppressor of the gray coat color of the *dilute* (Myo5a), *ashen* (Rab27a), and *leaden* (Mlph) mutants [9]–[11]. Myo5a, Mlph, and Rab27a act together as a motor complex to mediate melanosome transport in melanocytes of the skin [12], [13]. The gene encoding the *Mreg^dsu^* loss of function mutation has been identified as a vertebrate-specific, highly charged 214 amino acid protein, without functional domains or homology to other proteins, but containing a cysteine-rich N-terminal sequence suggestive of a membrane binding domain [1]. Melanoregulin functions in the melanosome transport system in the skin by influencing transfer of melanosomes from melanocytes to keratinocytes, presumably at the level of the plasma membrane or the melanosome membrane [1]. Recent studies suggest that melanoregulin may act as a cargo receptor in melanosome transport [14], although the identity of the cargo remains unknown.

Over a dozen genes are involved in HPS in mouse and at least seven orthologs have been identified in human. Many of these genes have recently been cloned [15]–[24]. With the exception of members of the adaptor protein (AP)-3 and HOPS complexes known to be involved in vesicle transport, most HPS genes encode vertebrate-specific proteins with ubiquitous expression and without homology or obvious functional domains. Electron microscopy studies of skin melanocytes in the various mouse mutants of HPS have yielded clues as to which genes are required for various stages of melanosome biogenesis; mutants of genes that act together have similar phenotypes [25], [26]. Yeast two hybrid and co-immunoprecipitation studies have confirmed the ultrastructural analyses in delineating the HPS proteins that interact in specific BLOC complexes, and showing that HPS3 (*cocoa*), HSP5 (*ruby2J*), and HPS6 (*ruby-eyed*) are components of BLOC-2 [22], [27], [28]. HPS3 (*cocoa*), a member of the BLOC-2 complex, binds to clathrin-coated vesicles in the cytoplasm of human skin melanocytes [29].

Cytoplasmic transport vesicles appear to be a key component of organelle, specifically melanosome, biogenesis. Melanogenic proteins are transported to melanosomes using a variety of methods, including the AP-3 and AP-1 pathways [30], and arrive at different stages in melanosome development. For example, tyrosinase relocates from the late endosome directly to stage II melanosomes, while Pmel17/gp100/silver is transported from AP-3 vesicles to stage I melanosomes, skipping endosomes altogether [31]. It is possible that HPS genes of unknown function are involved in the transport of other components of nascent melanosomes.

Like the HPS genes, Oa1/OA1 was identified by positional cloning [32]–[34] and displays structural characteristics typical of transmembrane G-protein coupled receptors [35]. Further studies have shown that OA1 indeed functions as an intracellular G-protein coupled receptor in both *Saccharomyces cerevisiae* [36] and mammalian cells [37], and L-DOPA was suggested as a ligand for OA1 but the signal transduction mechanisms triggered by L-DOPA is still unclear [38]. OA1 is localized at the membrane of melanosomes at all stages of maturation but higher amounts are found in melanosomes at early stages of maturation (stage I and II) and in late endosomes [39]. Because no spontaneous mutant exists for Oa1, likely due to the lack of an observable phenotype, a knockout mouse was created to study its function [40]. This mouse has features of human ocular albinism (OA1), including the macromelanosome in pigment epithelium and skin melanocytes a reduction of melanosome number and a reduced uncrossed retinal projection [40] [41], similar likewise to humans with oculocutaneous albinism and HPS, and similar to other hypopigmented mouse strains such as *Oca1^Tyr<c>^*, *Hps6^ru^*, *Hps2^pe^*, and *Hps1^ep^* [42], [43]. Although hypopigmented individuals with RPE melanosomal abnormalities from many genetic causes share the same visual system abnormalities, a common mechanism among these disorders is lacking.

We here provide the first evidence for a molecule that links the HPS and Oa1 pathways in melanosome biogenesis, and provide evidence that modulating the levels of melanoregulin can partially correct the melanosomal defects in the HPS BLOC-2 mutants *Hps6^ruby^*, *Hps5^ruby2J^*, and *Hps3^coa^*, and in the *Oa1* knockout mouse.

## Results

### Melanoregulin affects eye color of HPS mutants in a dose-dependent manner

**Figure 1:**
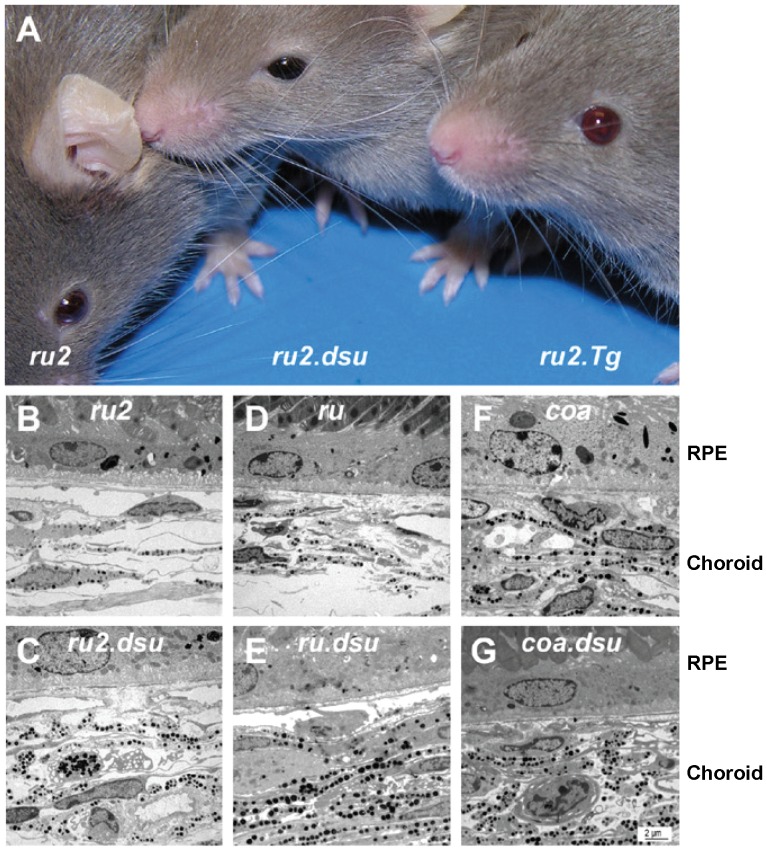
Melanoregulin levels modulate the eye color of HPS BLOC-2 mutant mice. A: Three month-old male mice of the genotypes indicated were photographed in ambient light. Note nearly black color of eyes of *Hps5^ruby2J^.Mreg^dsu^* mice, darker than the eyes of *Hps5^ruby2J^* mice, which are darker than those of the light ruby red *Hps5^ruby2J^.Mreg^Tg^* eyes. B–G: Electron micrographs of sections through the RPE and choroid of *Hps6^ruby^*, *Hps5^ruby2J^*, and *HPS3^coa^* mice, showing the lighter eye color in (A) to be secondary to decreased pigmentation of these layers. Note the effect of a melanoregulin loss of function (*Mreg^dsu^*) allele in enhancing pigmentation of the choroid in all three BLOC-2 mutants, *Hps6^ruby^*, *Hps5^ruby2J^*, and *Hps3^coa^*. Scale bar = 2 µm.

Melanoregulin loss of function suppresses the eye color defect in *ru2J (Hps6^ru2J^)*, *ruby (Hps5^ruby^)*, and *cocoa (Hps3^cocoa^)* mice from dark red to black; overexpression of wildtype melanoregulin lightens the eye color of these same HPS mutants to red, without affecting coat color (ruby2 shown in Figure 1A). Melanoregulin loss of function rescues the eye color in *ru2J*, *ruby*, and *cocoa* mice by increasing pigmentation of the choroid (Figure 1B–G). Mice carrying the *melanoregulin* loss of function (*Mreg^dsu^*) mutation on a wildtype background, transgenic mice overexpressing the wildtype *melanoregulin* gene (*Mreg^Tg^*), and *Oa1* knockout mice have no visible eye or coat color defects alone or in combination; all have black eyes and a black coat on a C57BL/6 background (data not shown).

### Loss of melanoregulin increases while overexpression decreases pigment incorporation into melanosomes of the RPE

**Figure 2:**
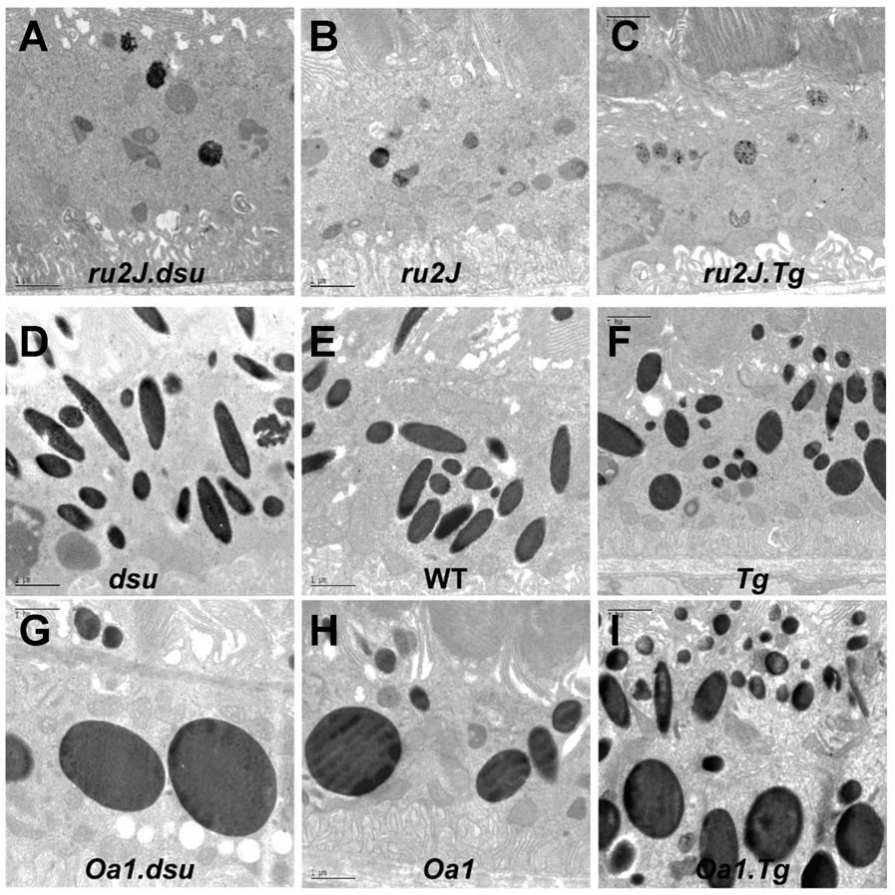
Size, shape, and density of melanosomes in the RPE of *Hps* and *Oa1* loss of function mouse mutants are altered by loss or overexpression of melanoregulin. A–I: Electron microscopic images of the RPE in mice of the genotypes indicated. Orientation is with photoreceptor outer segments toward the top and Bruch's membrane/choroid toward the bottom of each image. Abbreviations: *ru2J = Hps5^ru2J^; dsu = Mreg^dsu^; WT = wildtype; Tg = Mreg^Tg^ ; Oa1 = Oa1^ko^.* Scale bar = 1 µm.

**Figure 3:**
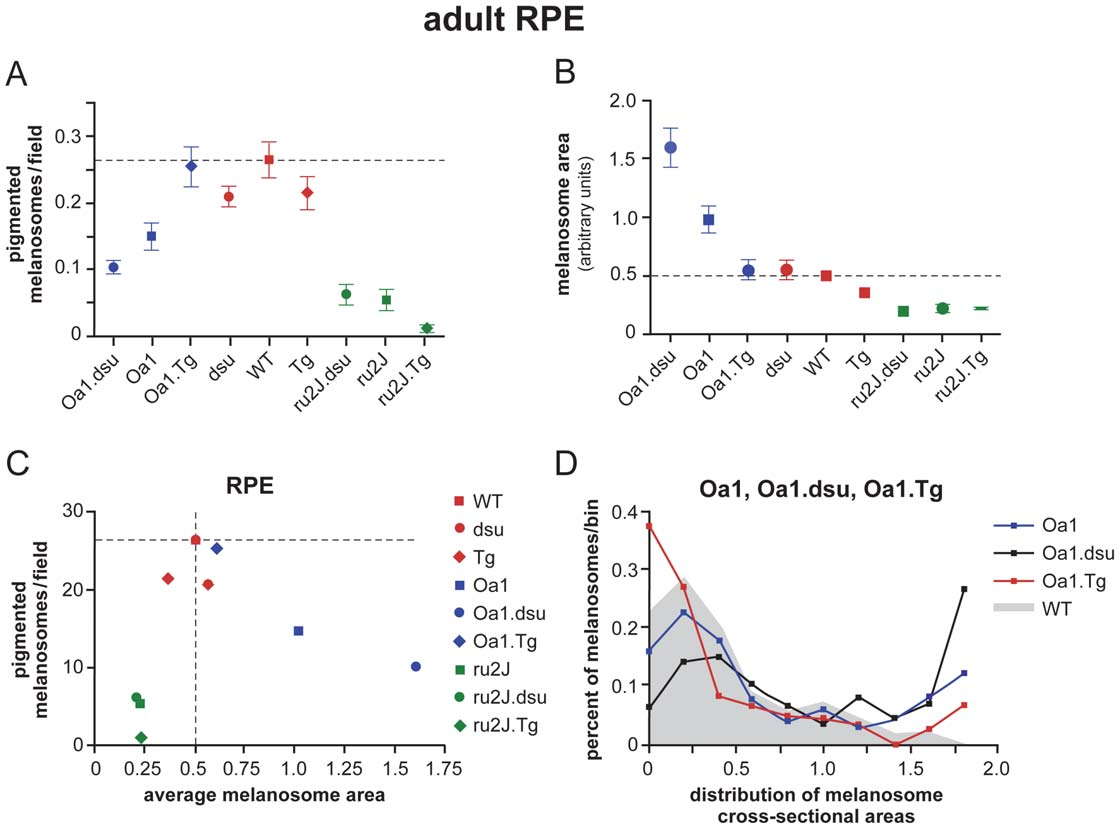
Melanosome density, cross sectional area, and size distribution exhibit a dosage-dependent effect of melanoregulin in the *Oa1* null mouse. Quantitation and analysis of the data shown in Figure 2. Data points are mean values ± SEM. Note the nearly inverse linear relationship between melanoregulin expression and A. Melanosome number and B. Melanosome area on an Oa1 null background. On an *Oa1* background, both melanosome number and area show a statistically significant change as a function of melanoregulin expression, with p<0.05 as indicated by asterisks (*). No statistically significant difference was observed in *Hps5^ru2J^* mice, p>0.05. *Mreg^dsu^* mice show a significant decrease in number of melanosomes, p<0.05 with no change in melanosome area, p>0.05. . C. The number of pigmented melanosomes per field is compared to the average melanosomal area in wildtype (C57BL6/J) mice. Abbreviations: *ru2J = Hps5^ru2J^; dsu = Mreg^dsu^; WT = wildtype; Tg = Mreg^Tg^; Oa1 = Oa1^ko^.*

In wildtype retinal pigment epithelium, melanosomes constitute a major component of the cytoplasm, ranging in size and cross-sectional shape from round to elongated. The width of both long and round melanosomes is consistently ≤0.5 µm (Figure 2E). In melanoregulin loss of function, (labeled dsu), little change is observed in the cross-sectional area, shape, or size distribution of melanosomes (Figure 2D), while the average density of melanosomes is slightly decreased as quantified in Figure 3A. By contrast, overexpression of melanoregulin via a BAC transgenic on a wild type background, (labeled Tg) results in a small decrease in both the average cross-sectional area of RPE melanosomes and density (Figures 2F and 3A, B). Overall, either loss or overexpression of melanoregulin has only minor consequences for melanosome size, shape, and density on an otherwise wildtype background (Figure 2D–F and Figure 3A–C).

Lack of HPS5 expression results in a drastic decrease in number, size, and pigmentation of RPE melanosomes in the *ru2J* mouse (Figures 2B, 3A). By contrast, loss of Oa1 expression results in a dramatic increase in the cross-sectional area of many RPE melanosomes, while others fall within the wildtype size range (Figure 2H). The average size and pigmentation of these melanosomes is greatly increased relative to wildtype, while average number is correspondingly decreased (Figure 3A,B). The normal elongated shape of melanosomes is strikingly lost in the *Oa1^ko/ko^* mice. While melanosomes vary in size and longest diameter in approximately the same range as wildtype, very few of the melanosomes are elongated. Those that reach a diameter similar to the longest diameter in wildtype mice are almost invariably round (Figure 2H).

To evaluate how melanoregulin contributes to melanosome biogenesis, we generated mice carrying *Hps5^ru2J^* or *Oa1^ko/ko^* alleles in combination with either two loss of function or two overexpression alleles of melanoregulin. In the RPE of *Hps5^ru2J^.Mreg^dsu^ (ru2J.dsu)* double mutant mice, melanosomes are the same size, shape, and density as in *ru2J* mice; however, some melanosomes appear to be more heavily pigmented (Figures 2A vs. 2B; 3A vs. 3B). In *Hps5^ru2J^.Mreg^Tg^ (ru2J.Tg)* mice, we again find little effect on melanosome size or shape; however, the number of pigmented melanosomes is negligible (Figures 2C vs. 2B; 3A,B).

Similarly, in *Oa1^ko/ko^* mice, loss of melanoregulin function results in a decrease in melanosome number along with an increase in size of the macromelanosomes compared to Oa1 loss of function alone (Figure 2G vs. 2H; 3A,B). As predicted from the results presented thus far, overexpression of melanoregulin decreases the average cross-sectional area of the largest melanosomes and also results in smaller than normal melanosomes (Figure 2I vs. 2H, 2E; 3A,B). In some cells, as shown in Figure 2I, melanoregulin appears to act in a graded fashion, with a stronger influence on melanosome size in the apical portion of the cell and a weaker influence on basal melanosomes.

Together these results show a consistent association between MREG expression and melanosome biogenesis in the RPE, whereby loss of melanoregulin function facilitates pigment incorporation while overexpression results in reduced melanization. This function of melanoregulin is most pronounced in the presence of another mutation that it either suppresses or enhances, or both, depending on the level of melanoregulin and the function of the other gene. In the case of *Hps5^ru2J^*, loss of melanoregulin has little effect, whereas overexpression of melanoregulin exacerbates the lack of melanosome pigmentation (Figure 3C). Conversely, on an *Oa1^ko/ko^* background, melanoregulin loss of function greatly worsens the macromelanosome phenotype, while overexpression almost completely rescues the average size and number of RPE melanosomes (Figure 3C).

Because the size distribution of melanosomes in *Oa1.Tg* RPE appears more uneven than in wildtype RPE, the distribution of melanosome cross-sectional areas was plotted for each of the Oa1-related mutants compared to wildtype (Figure 3D). Based on this view of the data, *Oa1^ko/ko^;Mreg^Tg^* (*Oa1.Tg*) is shown to have a greater fraction of small melanosomes and also more large melanosomes than wildtype; however, the distribution is closer to wildtype in having a smaller fraction of macromelanosomes than either *Oa1^ko/ko^* alone or *Oa1^ko/ko^;Mreg^dsu^* (*Oa1^ko/ko^.dsu*). The size distribution for melanoregulin loss of function or overexpression alone does not vary significantly from wildtype (not shown; Figure S1).

### Melanosome defects in the choroid of HPS5^ru2J^ mice are rescued by loss of melanoregulin function

**Figure 4:**
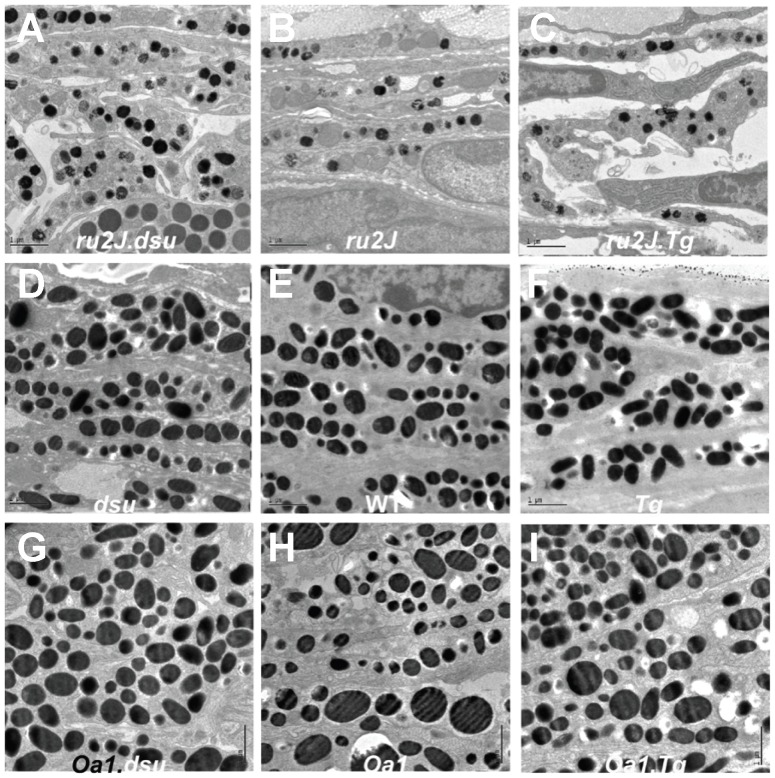
Loss of melanoregulin function partially rescues melanosome size and density in the choroid of *Hps5^ru2J^* mutant mice. A–I: Electron microscopic images of the pigment choroid layer in mice of the genotypes indicated. Orientation is with RPE toward the top of each image. Abbreviations: *ru2J = Hps5^ru2J^; dsu = Mreg^dsu^; WT = wildtype; Tg = Mreg^Tg^ ; Oa1 = Oa1^ko^.* Scale bar = 1 µm.

**Figure 5:**
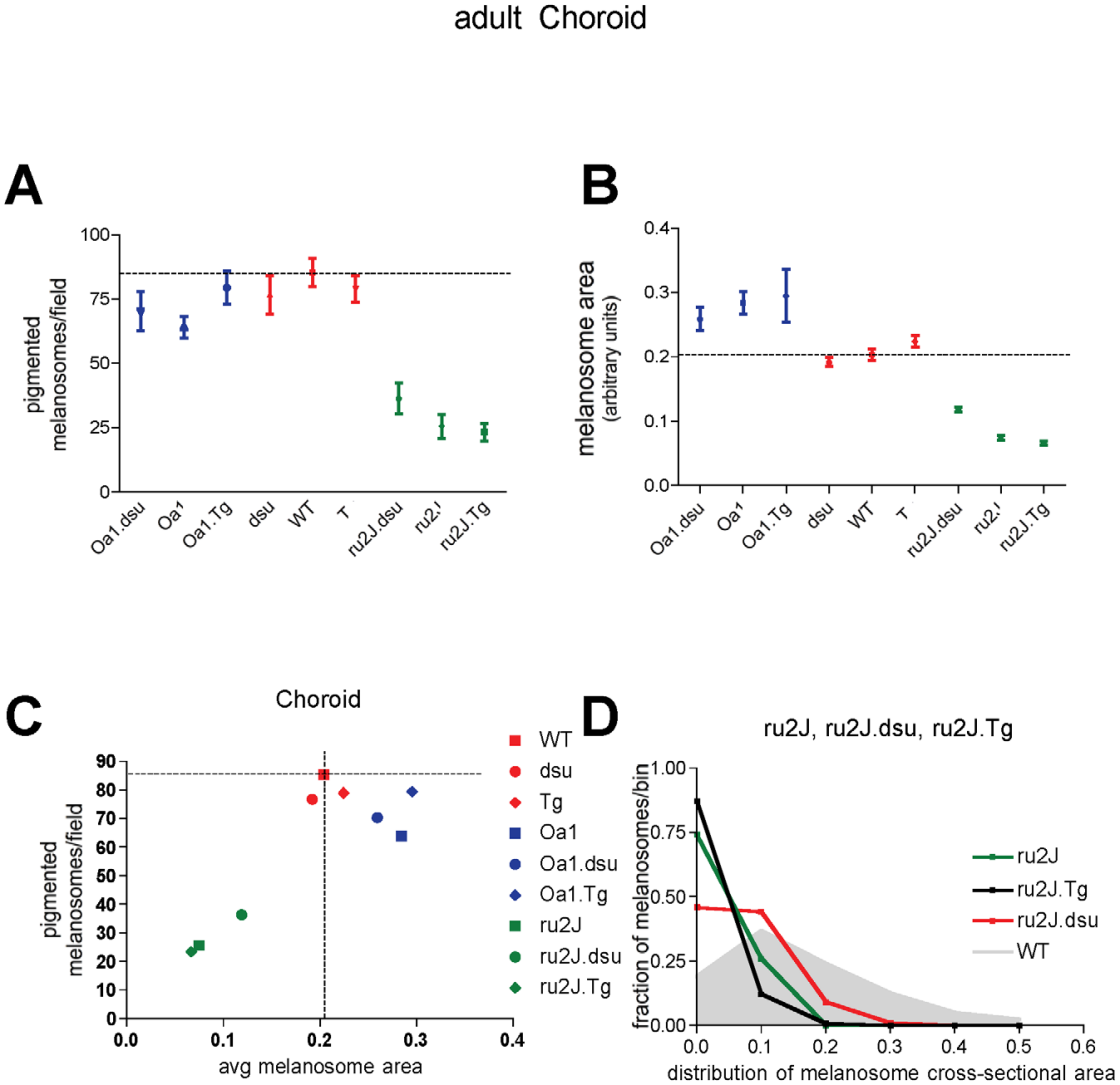
Melanoregulin levels influence melanosome characteristics in the choroid of *Hps5^ru2J^* and *Oa1* mutant mice. Quantitation and analysis of the data shown in Figure 4. Data points are mean values ± SEM. A. Melanosome number and B. Melanosome area on an *Oa1* null background do not show a statistically significant change as a function of melanoregulin dose; all p values are p>0.05. In contrast, in *Hps5^ru2J^* mice, both melanosome number and area increase, p<0.05. *Mreg^dsu^* mice show a significant increase in melanosome area, p<0.05, with melanoregulin over-expression (*Mreg^Tg^*) and a decrease from wildtype in melanoregulin loss of function mice (*Mreg^dsu^*), p<0.05, with no significant change in number of melanosomes in either case, p>0.05. C. The number of pigmented melanosomes per field is compared to the average melanosomal area in wildtype (C57Bl6/J) mice.

The melanosomes in the choroid of wildtype mice are on average less than half the size (measured as cross-sectional area) of RPE melanosomes and have a smaller size distribution and higher density per cell (Figures 4E and 5A,B). Neither loss nor overexpression of melanoregulin has a substantial effect on size, shape, or density of choroidal melanosomes on an otherwise wildtype background (Figures 4D,F and 5A,B). In the *HPS5^ru2J^* mutant, the number and size of choroidal melanosomes are significantly retarded (Figures 4B and 5A,B); melanoregulin overexpression has almost no effect (Figures 4C and 5A,B), while melanoregulin loss of function results in a significant increase in both the size and number of pigmented melanosomes (Figures 4A and 5A,B). In *Oa1^ko/ko^* mice, the situation is a mirror image of HPS, where loss of melanoregulin has little effect on size or density of choroidal melanosomes while overexpression results in a slight rescue of melanosome density (Figures 4H,I,J and 5A,B). Overall, the results point to an effect of melanoregulin loss of function facilitating pigment incorporation into maturing melanosomes in the choroid.

The effects of melanoregulin expression on melanosome biogenesis in the choroid are not as pronounced as in the RPE, in that all three Oa1 mutants are more similar to each other than to melanoregulin loss or overexpression on a wildtype background (Figure 5C). Even the rescue in *HPS5^ru2J^.dsu* leaves this mutant with more similarity to *HPS5^ru2J^* or *Hps5^ru2J^.Tg* than to wildtype in terms of both number and size of melanosomes (Figure 5C). Most prominently, an evaluation of the size distribution of melanosomes in the *Hps5^ru2J^* mutants reveals a significant correction in the range of melanosome sizes in *Hps5^ru2J^.dsu* compared to either *Hps^ru2J^* or *Hps5^ru2J^.Tg* (Figure 5D), which is enough to result in significant coloration of the eye (Figure 1A).

### Melanoregulin protein localizes to cytoplasmic vesicles in the RPE

**Figure 6:**
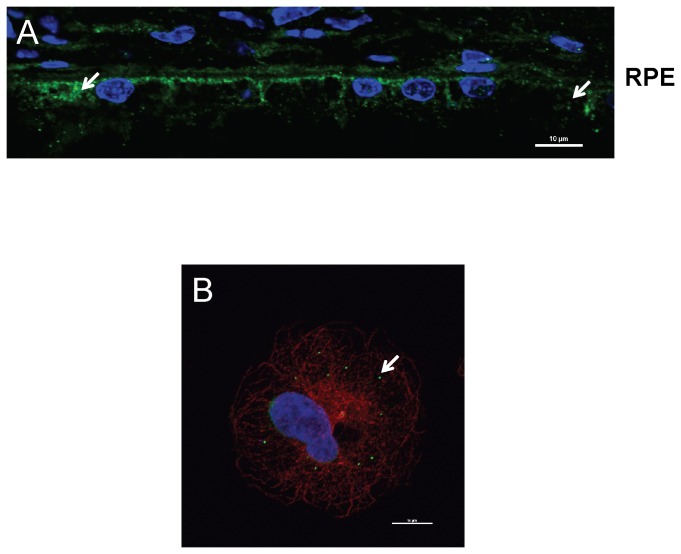
Melanoregulin protein localizes to vesicle-like structures in the RPE cytoplasm. A: Frozen 7 um sections of 4-month-old mouse retinas were stained with anti-Mreg antibody. Nuclei were visualized with DAPI. Images were captured on a Nikon A1R live cell confocal imaging system. B: ARPE19 cells were fixed and stained with anti-MREG mouse mAb, (Novus Biologics) 1∶250, anti-alpha tubulin rabbit pAb (Thermo) 1∶200, washed and incubated with secondary antibodies: Alexa Fluor 488 donkey anti- mouse IgG (1∶1000), Alexa Fluor 594 donkey anti-rabbit (1∶1000) and Hoechst 33258 (1∶10,000). Images were captured on a Nikon A1R laser scanning confocal microscope with a 100× oil objective and processes using Nikon's Elements software.

Melanoregulin is distributed in the basolateral RPE of wild type mice as shown in Fig. 6A. Within individual RPE cells melanoregulin is localized to small cytoplasmic vesicle-like structures less than 100 µm in diameter (Figure 6B) and [44]. This pattern is similar to that found for transfected HPS3-GFP, recently found to colocalize with cytoplasmic, clathrin-coated vesicles in cultured skin melanocytes [29].

## Discussion

**Figure 7:**
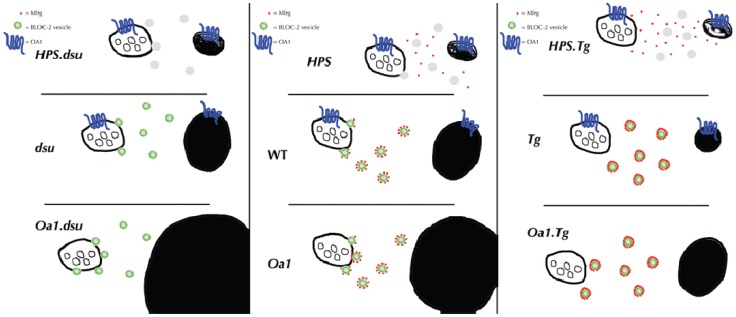
Model of melanoregulin function in melanosome biogenesis. These results are consistent with a hypothetical and testable model in which melanoregulin functions as a negative regulator of vesicle fusion with cytoplasmic components required for melanosome biogenesis. The panels in the diagrams are analogous to the panels in Figures 2 and 4, and show both a multivesicular body/endosome and a mature/pigmented melanosome as two stages in the biogenesis of melanosomes. HPS BLOC-2 forms a putative vesicle transport complex, or part of a vesicle system required for moving proteins required for melanosome biogenesis. Melanoregulin acts as a negative regulator of membrane fusion between BLOC-2 and the late endosome/early melanosome. Oa1 acts as a permissive gatekeeper, allowing components of melanosome biogenesis to accumulate up to a certain point, at which time it initiates (or turns off) a signalling cascade that prevents further accumulation of pigment-producing components. These features are compatible with the results presented here, and predict the results of future experiments that can be performed to test the tenets of this model.

The reciprocal relationship between the level of melanoregulin expression and melanosome size suggests that this protein may act as a negative regulator of membrane fusion, either directly in the fusion process or indirectly by facilitating the transport of vesicles (Figure 7). This interpretation is consistent with the findings that *dsu* loss of function alone has no grossly visible phenotype, and that overexpression results in a decrease in melanosome size without loss of pigmentation. While other forms of albinism affect both skin (hair follicles) and retinal pigment, the effects of MREG and OA1 appear to be primarily restricted to the eyes. Changes in melanosomes do occur in hair follicles as well as in eyes of *Mreg^dsu^* mutants (R. Rachel, unpublished results). However, these changes are less clear because the alterations in hair follicles involve not only melanogenesis but also incorporation of melanin packets into the hair shaft. As such, a specific effect on melanogenesis is not quantifiable.

Melanoregulin could function in concert with BLOC complexes transporting vesicles to the early melanosome, by preventing these transport vesicles from fusing with developing organelles under certain conditions, e.g., melanosome size or levels of other proteins. Melanoregulin may serve as a linker molecule between the BLOC-2 complex and Oa1, which has been shown to regulate melanosome protein trafficking at early stages of melanosome biogenesis [39]. Localization of Melanoregulin in cytoplasmic vesicles may suggest an interaction with Oa1, which regulates correct targeting of melanosomal protein loaded vesicles to proper stages of melanosomes during their maturation. This hypothesis is supported by a series of findings showing a role for OA1 in melanosome biogenesis via vesicle budding or fusion in the late endosomal compartment [45]–[47]. MREG has been localized to Syn-7 and LAMP-2 rich late endosomal compartments, consistent with a role in this process [44]. In fact, OA1 loss of function causes mislocalization of melanosomal protein Tyrp1 in stage I/II melanosomes causing melanin deposit in not yet mature organelles [39]. This interpretation is consistent with the recent observation that Melanoregulin interacts with RILP (*p150^glued^*) [14].

These findings have potential implications for treatment for HPS and OA1 in that inhibition or enhancement of melanoregulin, respectively, may be beneficial in modulating the ocular pigment phenotype of these individuals. What remains to be determined is the extent to which modulating the pigment phenotype in each disorder will correct the visual system defects that accompany it. Interestingly, the melanosomes in both HPS and OA1, irrespective of being undersized or oversized, cause similar defects in the neural retina to complete lack of pigment due to loss of the melanogenic enzyme tyrosinase (for review, see [7]); this suggests that the melanosome is required for signaling to the neural retina and that normal melanosome function is important for this signaling, not just the presence or absence of pigment [48]. This places the melanosome in a more dynamic role: that of active participant in the function of the RPE, rather than being a passive medium for light absorption. A polyacetylene, melanin in intact melanosomes has been shown to exhibit semi-conductor properties, emitting a flash of light when it switches from low to high conductivity [49]. This feature suggests that the conversion of electrical energy to heat or light may provide a novel mechanism for activation or deactivation of biological processes. Generalizing the findings with HPS and melanoregulin linking to OA1 as a size sensor in melanosome biogenesis, we might expect other G-protein coupled receptors to play similar roles in the formation of other intracellular organelles.

## Experimental Procedures

### Materials

Commercially available antibodies were purchased as follows: anti-MREG mouse mAb, (Novus Biologics) 1∶250; anti-alpha tubulin rabbit pAb (Thermo) 1∶200; AlexaFluor 488 donkey anti- mouse IgG (1∶1000); AlexaFluor 594 donkey anti-rabbit (1∶1000) and Hoechst 33258 (1∶10,000).

### Generation of mouse lines

*dsu/dsu*, *d^v^/d^v^* mice [11] were obtained from Jackson Laboratories and have been maintained at NCI-Frederick for 90+ generations. To obtain mice carrying only the *dsu* mutation, *dsu/dsu*, *d^v^/d^v^* mice were backcrossed to C57Bl/6J for 10 generations, followed by intercrossing and selecting for mice wildtype at the *dilute* locus and carrying *dsu/dsu*. Some of the data shown here derives from mice homozygous for *dsu* at the N5 backcross generation while the rest is from the N10 backcross line. Crosses between *dsu* and *ru2J* were carried out with *B6.dsu* mice at the N5 generation, while crosses to Oa1 were done with B6.*dsu* mice at the N10 generation. Mice were genotyped using the following primers: Primers for *d^v^*: Right, common b7: TCCTCTGTGGTCATCACTGG, Left, wildtype: TGGAATCCCAGCAGTGGTA, 179 bp product. *d^v^*, Left, viral allele: CCCGTGTATCCAATAAAGCC, 198 bp product. Primers for *dsu*: Right, common dsu5′brk-f27 CTGGGAGTTCAAGGTTGGTCTG, Left wildtype, dsu5′brk-b24 GCAGGAGAGGCTGGGAAAAAAC, 230 bp product wildtype, Left dsu, dsu3′brk-b CCACAGTCTCAAGTCTTTCCTG, dsu ∼140 bp. Transgenic mice overexpressing wildtype *dsu* (in the text, Tg) were generated as described [1]. *dsu* and Tg alleles were separately bred onto lines of mice carrying either *ru^2J^ (HPS5)*, *ru (HPS6)*, or *coa (HPS3)* mutations, or onto the B6. *Oa1^ko/ko^* knockout line. Ru2J/ru2J mice were selected based on coat color, while *Oa1^ko/ko^* mice were genotyped according to [40]. Mice were housed under cyclic light conditions: 12-h light/12-h dark and fed ad libitum. All procedures involving animals were in accordance with institutional ACUC-approved protocols and with the Association for Research in Vision and Ophthalmology (ARVO) guidelines for use of animals.

### Electron microscopy

Standard procedures for thin-section transmission electron microscopy (TEM) are previously described in detail [50]. A few minor modifications of the procedure were used for mouse eyes. Adult mice were perfused with 0.1 M phosphate buffered saline followed by 2% glutaraldehyde/4% paraformaldehyde fixative in cacodylate buffer (0.1 M, pH. 7.4). The eyes were dissected out. After post-fixation, the cornea and lens were removed, marking the orientation with a slit at the ventral pole. Eyes were rinsed thoroughly with cacodylate buffer and post-fixed in 1% osmium for 1 hr at room temperature, then dehydrated in graded ethanol (e.g., 35%, 50%, 70%, 95%, and 100%) and propylene oxide (100%). Eyes were infiltrated overnight in a 1∶1 mixture of propylene oxide and epoxy resin and embedded in pure resin and cured at 55°C for 48 hrs. The cured block was trimmed and made semi-thin sections (0.5 µm) and stained in Toluidine Blue-O solution to determine proper orientation for thin-section EM analysis. Thin-sections (60 nm) were mounted on naked 200-mesh copper grids, and stained in uranyl acetate and lead citrate. For measuring the number and size of melanosomes, RPE and choroid were imaged on a Hitachi H7000 (Tokyo, Japan) transmission electron microscope at 3000× and images obtained with a Gatan (Pleasanton, CA) digital camera and imaging software. For adults, counts were made on a 40 µm^2^ area on 10–20 images from each of 2–3 animals of each genotype. Counts on P5, P16, and P28 animals were done on 10 or more images from one animal of each genotype at each age.

### MREG localization in retinal sections and human ARPE19 cells

Immunohistochemistry was performed on frozen sections of 4-month-old mouse retinas. The eyecups were fixed in 4% paraformaldehyde in PBS (pH 7.4) overnight at 4°C, cryoprotected in 30% sucrose, and embedded in OCT. The 7 um sections were stained with anti-MREG mAb (Novus Biologicals) at 1∶200, washed 3× and stained with secondary antibody Alexa 488 anti mouse, dilution 1∶500. Controls were incubated with the secondary antibodies only. Nuclei were visualized with DAPI. Images were captured on a Nikon A1R live cell confocal imaging system. Data were analyzed using Nikon Elements AR Software 3.2.

ARPE19 cells grown on glass coverslips for 48 hours were rinse in PBS and then fixed and permeabilized in 4% buffered paraformaldehyde, with 0.2% Triton X100 for 10 minutes at room temperature. After three successive washes in PBS with 0.05%Triton X100 (PBST) the coverslips were blocked in PBST containing 4% Bovine serum albumin for 60 mins at 37°C. After blocking, coverslips were incubated at 37°(-include degree symbol)C for 60 minutes with primary antibodies: anti-MREG mouse mAb, (Novus Biologics) 1∶250, anti-alpha tubulin rabbit pAb (Thermo) 1∶200. After the incubation with primary antibodies, the coverslips were washed 3× in PBST and then incubated with secondary antibodies: Alexa Fluor 488 donkey anti-mouse IgG (1∶1000), Alexa Fluor 594 donkey anti-rabbit (1∶1000) and Hoechst 33258 (1∶10,000) at 37°C for 60 minutes. Three successive PBST washes followed the incubation with secondary antibodies. In preparation for microscopy, the coverslips were mounted in Cytoseal (Electron Microscopy Sciences). Images were captured on a Nikon A1R laser scanning confocal microscope with a 100× oil objective and processes using Nikon's Elements software.

### Statistical Analysis

Data are reported as the mean ± SEM with statistical analysis using a one-way ANOVA with appropriate post-hoc test. Results with a p<0.05 were considered significant. Significance of analyses is indicated in the figure legends.

## Supporting Information

Figure S1 **Melanoregulin loss of function or overexpression have little effect on the size distribution of melanosomes in either the RPE or choroid.** Data quantitated as shown in Figures 3D and 5D. The shape of the curves is nearly identical in wildtype, melanoregulin loss of function, and in melanoregulin overexpression. Data shown are an average ± SEM; differences are not statistically significant with p>0.05.
(TIFF)
