# Supplementary figures and images for: Melanoregulin, Product of the dsu Locus, Links the BLOC-Pathway and Oa1 in Organelle Biogenesis
Source: PLoS One. 2012 Sep 11;7(9):e42446. doi: 10.1371/journal.pone.0042446 (PMC3439427; doi:10.1371/journal.pone.0042446)

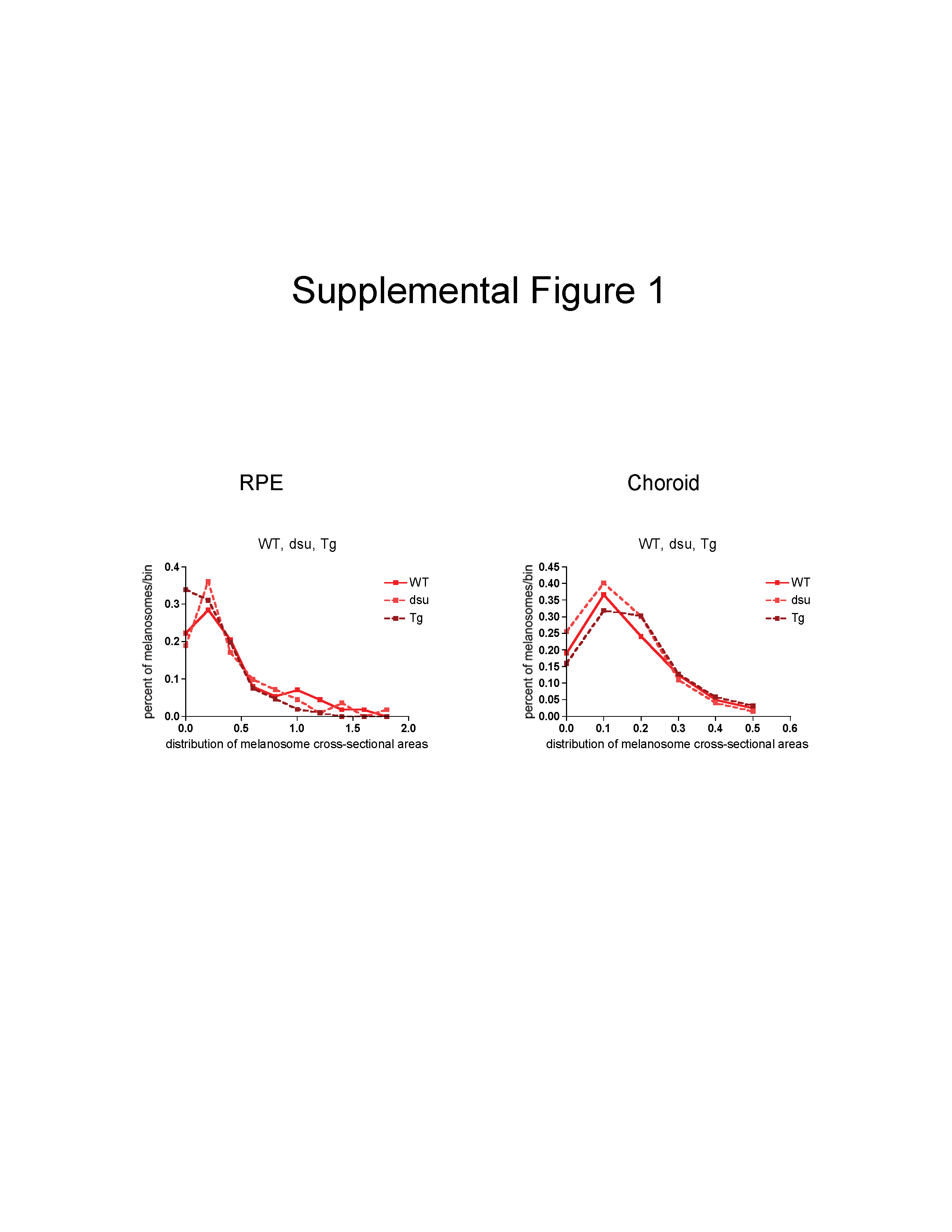

Supplement: Figure S1 — Melanoregulin loss of function or overexpression have little effect on the size distribution of melanosomes in either the RPE or choroid. Data quantitated as shown in Figures 3D and 5D. The shape of the curves is nearly identical in wildtype, melanoregulin loss of function, and in melanoregulin overexpression. Data shown are an average ± SEM; differences are not statistically significant with p>0.05. (TIFF) [file pone.0042446.s001.tif]
